# Web-Based Therapist Training in Interpersonal Psychotherapy for Depression: Pilot Study

**DOI:** 10.2196/jmir.7966

**Published:** 2017-07-17

**Authors:** Kenneth A Kobak, Joshua D Lipsitz, John C Markowitz, Kathryn L Bleiberg

**Affiliations:** ^1^ Center for Telepsychology Madison, WI United States; ^2^ Department of Psychology Ben Gurion University of the Negev Beer Sheva Israel; ^3^ Department of Psychiatry New York State Psychiatric Institute and Columbia University College of Physicians & Surgeons New York, NY United States; ^4^ Weill Cornell Medicine Department of Psychiatry New York, NY United States

**Keywords:** psychotherapy, Internet, depression, education, humans, computer-assisted instruction

## Abstract

**Background:**

Training mental health professionals to deliver evidence-based therapy (EBT) is now required by most academic accreditation bodies, and evaluating the effectiveness of such training is imperative. However, shortages of time, money, and trained EBT clinician teachers make these challenges daunting. New technologies may help. The authors have developed the first empirically evaluated comprehensive Internet therapist training program for interpersonal psychotherapy (IPT).

**Objective:**

The aim of this study was to examine whether (1) the training protocol would increase clinicians’ knowledge of IPT concepts and skills and (2) clinicians would deem the training feasible as measured by satisfaction and utility ratings.

**Methods:**

A total of 26 clinicians enrolled in the training, consisting of (1) a Web-based tutorial on IPT concepts and techniques; (2) live remote training via videoconference, with trainees practicing IPT techniques in a role-play using a case vignette; and (3) a Web-based portal for therapists posttraining use to help facilitate implementation of IPT and maintain adherence over time.

**Results:**

Trainees’ knowledge of IPT concepts and skills improved significantly (*P*<.001). The standardized effect size for the change was large: *d*=2.53, 95% CI 2.23-2.92. Users found the technical features easy to use, the content useful for helping them treat depressed clients, and felt the applied training component enhanced their professional expertise. Mean rating of applied learning was 3.9 (scale range from 1=very little to 5=a great deal). Overall satisfaction rating was 3.5 (range from 1=very dissatisfied to 4=very satisfied).

**Conclusions:**

Results support the efficacy and feasibility of this technology in training clinicians in EBTs and warrant further empirical evaluation.

## Introduction

The importance of preparing mental health professionals to deliver evidence-based therapy (EBT) is now well established [1]. Accreditation bodies for academic programs in psychiatry [2], psychology [3], and social work [4] in both the United States and Canada [5] require demonstration of competence in EBTs [6]. This requirement helps to address the critical shortage of clinicians trained in EBTs, which has been identified as a major public health concern [7,8]. It also accords with the needs both of patients, who generally prefer talk therapy to medication [9], and trainees, who report wanting to spend more time delivering EBTs and to receive more EBT training than they now do [10].

Interpersonal psychotherapy (IPT) is one of the oldest and best-studied EBTs [11,12] and one of the psychotherapies formally evaluated for efficacy by the National Institute for Mental Health (NIMH) [13]. Numerous professional and international guidelines recommend IPT for the treatment of major depression, including the American Psychiatric Association [14] and the Guidelines for Primary Care Physicians [15-17]. IPT focuses on understanding the interpersonal and social context in which the patient’s symptoms arose. This brief, time-limited approach focuses on feelings, validating them in the context of social situations, and helping the patient to understand and verbalize those feelings to change interpersonal encounters. A recent meta-analysis of 90 randomized controlled trials involving over 11,434 patients found large effect sizes for IPT in alleviating major depression. IPT was also effective in preventing relapse and had a preventive effect on subthreshold episodes [18]. Effect sizes were comparable with those found with cognitive behavior therapy (CBT) [19]. IPT has also demonstrated efficacy for other diagnoses including eating and anxiety disorders and for patients ranging from adolescents to the elderly [20]. Despite the substantial empirical literature supporting the efficacy of IPT, it has been far less disseminated than CBT.

With the increasing emphasis on EBTs comes an increasing focus on how to implement and evaluate psychotherapy training. Accreditation bodies in psychiatry (Accreditation Council for Graduate Medical Education) and psychology (American Psychological Association) require not only training in EBTs but an evidence-based approach to evaluating the effectiveness of such training, that is, whether clinicians demonstrate competence in administering the treatment [3,6]. Thus, psychotherapy training involves the dual challenges of expanding the current curriculum to include EBTs and ensuring such training is effective. These shortages of time, money, and availability of trained EBT clinician teachers make these challenges daunting. The development of new methods in psychotherapy training has been suggested as one solution to these challenges [21].

New technologies may help [22]. Internet-based training (e-learning) is cost-effective, scalable, and available upon demand. It overcomes limitations of trainer availability, especially in remote locations. Clinicians can work at their own pace, repeating and reviewing as desired. Enhancing training quality are the standardization of instruction (ensuring inclusion of key empirically proven components) and the use of multi-modal learning techniques the technology affords, which have been shown to increase knowledge uptake and retention [23]. Research has found Internet-based training has greater effectiveness than paper-based manuals alone, resulting in greater long-term knowledge retention [24,25], and in one study, Internet-based training was superior to face-to-face instruction [26]. Once the learner has mastered the didactic content, new technologies can teach and assess clinical proficiency in administering the newly learned skills. Both computer simulations using virtual patients [27,28] and remote live training via videoconference have demonstrated effectiveness in teaching applied clinical skills [29,30]. Posttraining, these same technologies can be used to help ensure proper implementation in clinical practice [22,31]. Research has found posttraining consultation an essential ingredient for successful implementation of EBT skills [32] and to predict clinician adherence and competence following EBT training [32,33]. By improving access to training, new technologies can help facilitate dissemination of a variety of EBTs, including IPT and provide patients with a wider choice of treatment options.

In this pilot study, we developed the first comprehensive Internet training program for IPT to be empirically evaluated. This three-part, interactive therapist training protocol focuses on IPT for major depression and consists of (1) a Web-based tutorial on IPT concepts and techniques; (2) live remote training via videoconference, with trainees practicing IPT techniques in a role-play using a case vignette; and (3) a Web-based portal for therapists posttraining use to help facilitate integration of IPT into their clinical practice and maintain adherence and quality over time. The goal of the study was to examine the following hypotheses: (1) the training protocol would increase clinicians’ knowledge of IPT concepts and skills from baseline and (2) clinicians would deem the training feasible as measured by satisfaction and utility ratings.

## Methods

### Procedure

Before training, trainees took a 38-item pretest on their knowledge of IPT concepts and principles. Following the pretest, they received a username and password to access the Web-based tutorial and completed it at their own pace. Trainees could email the instructors with questions about the material. After completing the Web-based tutorial, trainees took a posttest of IPT knowledge and a user satisfaction questionnaire. Trainees then received a 45-60 min live applied training session conducted via videoconference with an experienced IPT trainer (JDL, JCM, or KLB). During this session, the trainer portrayed a standardized depressed patient, whereas the trainee role-played as therapist (see below). After completing the video session, trainees completed a satisfaction questionnaire and received a link to the IPT posttraining website. The posttraining website was designed to facilitate implementation and adherence following training and to guide the clinician in structuring sessions with their first IPT patients.

### Description of Training Components

#### Web-Based Tutorial

The training components paralleled components of IPT. In IPT, the patient and IPT therapist together define a central interpersonal problem focusing on one of four categories: grief, role transition, role dispute, or interpersonal deficits [20]. IPT has three phases: the initial phase (evaluation, case formulation, and treatment plan), the middle phase (addressing and resolving the focal problem), and the termination phase (consolidating gains and transitioning from treatment). The tutorial contains seven modules covering these and other topics on the theory and practice of IPT for depression. A description of the content and learning goals for each of the modules is presented in Table 1.

**Table 1:** Learning objectives by module: interpersonal psychotherapy (IPT) tutorial.

Module	Learning goal
Welcome and overview	Describe the goals of the tutorial
Principles of IPT^a^ for depression	Describe the theoretical roots for IPT
	Describe the IPT theory for the cause and treatment of depression
	Describe the role of the IPT therapist Describe the role of the IPT patient given depression as a medical illness
	Describe the three phases of IPT treatment, the interpersonal inventory, and developing a case formulation
3: The four IPT problem areas: grief	Explain the difference between normal grief and abnormal grief (complicated bereavement)
	Describe the therapeutic goals in treating depression resulting from abnormal grief
	Describe questions to use in order to assess the presence of abnormal grief
	Describe how to facilitate the grieving process
The four IPT problem areas: role transition	Describe a role transition
	Describe how a role transition may result in depression
	Identify when a role transition is an issue for a patient
	Explain the treatment goals in treating depression resulting from a role transition
The four IPT problem areas: role dispute	Describe the nature of role disputes
	Identify when a role dispute is an issue for a patient
	Identify the three stages in role disputes
	Describe the therapeutic goals in treating patients presenting depression resulting from a role dispute
The four IPT problem areas: interpersonal deficits	Describe when interpersonal deficits are the focus of treatment
	Describe the treatment goals in IPT in treating depression resulting from interpersonal deficits
	Describe how patients with interpersonal deficits differ from patients presenting with depression resulting from the other three problem areas
Mechanisms of change in IPT	Describe the four ways in which IPT achieves therapeutic goals

^a^IPT: interpersonal psychotherapy.

Because multi-modal learning and high levels of interactivity enhance learning [23,34], content was presented in varied formats including animations, graphical illustrations, and clinical vignettes with audio and interactive exercises. New material was often presented using “challenge questions,” as learning is enhanced when a user makes mistakes applying recently acquired information and gets specific feedback explaining the rationale for the correct answer [35]. Presentation of material was guided by principles of instructional design, such as “chunking” material based on limits to working memory to enhance retention [36]. It was estimated the tutorial would take 3 to 4 hours to complete. Interactive demos of the tutorial content can be found in Multimedia Appendices 1 and 2.

#### Applied Training

After completing the Web-based tutorial and Web-based posttest, trainees completed a supervised clinical training session using videoconferencing. During the applied session, the trainer portrayed a standardized depressed patient, whereas the trainee role-played the IPT therapist. The goal was to offer trainees practice in applying the skills learned during the tutorial. The trainer provided feedback and suggestions in real time as appropriate. Trainees also had the opportunity to ask questions about the IPT approach. The role play was designed to portray the patient’s second or third session, to provide the trainee an opportunity to practice developing an interpersonal formulation with the patient, and achieving agreement with the patient on the focal IPT problem area. We did not role play initial sessions because these typically focus on history gathering, which we finessed by providing summary information on the patient’s history up front to the trainee. Starting a session with “How have things been since we last met?” is a pattern that begins with session 2. A crucial point in IPT treatment is the therapist’s formulation of a focal problem area in session 2 or 3 and getting the patient’s agreement on it; this then organizes the remainder of the treatment [37]. Hence, the choice of session 2-3 from a time-limited, 12-16 session acute treatment framework. Although not sufficient for advanced clinical training in IPT, the applied training allowed us to evaluate the feasibility and user satisfaction with this approach. Applied training where trainees actually implement the skills learned has been found a critical, and often lacking, component of training [38-41].

#### Posttraining Case Tracker Website

A limitation of many professional training programs is lack of carryover to practice [32]. To address this, after completing the applied training session, trainees received access to the interactive IPT website portal (IPT Case Tracker). The IPT Case Tracker was designed to facilitate the transition from classroom and initial applied training to clinical practice and to help maintain adherence to IPT in initial and ongoing clinical use. The portal contained (1) interactive tools (eg, reminders) to help clinicians structure their IPT sessions, assist with case conceptualization, and provide an overall framework for conducting IPT with specific clients; (2) checklists for presession preparation and postsession tracking of client issues, client progress, and utilization of IPT skills; (3) a printable depression measure (Hamilton Depression Rating Scale [42]) to monitor treatment progress; and (4) case consultation via email, to facilitate uptake and troubleshoot implementation questions and issues. We obtained user satisfaction with the case tracker 1 to 3 months after completion of the applied training session. See Multimedia Appendices 3 and 4 for samples of pre- and postsession checklists.

### Measures

#### Efficacy

To assess trainees’ gains in knowledge of the concepts, principles, and techniques of IPT, the authors developed a 38-item pre- and posttest covering the core concepts in the tutorial. The text contained a combination of multiple-choice and true-false questions in proportions mandated by continuing education guidelines from the American Psychological Association and the National Association of Social Work. Testing served dual functions of assessing and reinforcing learning. Trainees received rationales for the correct answers after completing the posttest to reinforce learning. Posttests were given after completion of each module. The test had good internal consistency reliability (coefficient alpha =.79).

#### Feasibility

We evaluated user satisfaction from two perspectives: technical implementation, and clinical content. User satisfaction with technical aspects of the training tutorial was assessed using the System Usability Scale (SUS) [43,44], a reliable, well-validated 10-item scale designed to evaluate user satisfaction with technical aspect of Web-based applications and other technologies. It provides a score on a 0-100 scale regarding the effectiveness, efficiency, and satisfaction users experience using the system. A mean SUS score of 50.9 represents a rating of “okay,” 71.4 represents a rating of “good,” 85.5 represents a rating of “excellent,” and 90.9 represents a rating of “best imaginable.”

Descriptive statistics assessed trainee satisfaction with the clinical content of the training components. Trainees rated each training component along six dimensions using a 4-point scale (strongly agree, agree, disagree, and strongly disagree). Trainees also rated global satisfaction and had opportunity for open-ended feedback. Scale items were developed in prior studies on user satisfaction with Web-based training [29,45] and have shown good internal consistency reliability (Cronbach alpha=.92). Ratings on the extent to which trainees felt the learning goals were met and global ratings of how much was learned were also obtained for the tutorial as another measure of feasibility.

### Statistical Analyses

A two-tailed paired *t* test was computed to examine the mean change knowledge assessment from pretest to posttest on the Web-based tutorial. The standardized mean effect size for the *t* test was calculated using methods described by Cohen [46]. Effect sizes were considered large at 0.80, medium at 0.50, and small at 0.20 [46]. Descriptive statistics were used for the feasibility measure results.

### Compliance With Ethical Standards

This study has been approved by the Allendale Institutional Review Board. All procedures performed in this study involving human participants were in accordance with the ethical standards of the institutional and/or national research committee and with the 1964 Helsinki declaration and its later amendments. Informed consent was obtained from all individual participants included in the study. This article does not contain any studies with animals performed by any of the authors.

## Results

### Participants

Clinicians were recruited through advertisements in professional journals from National Association of Social Work and the American Psychological Association and through an announcement on the International Society for Interpersonal Psychotherapy (ISIPT) listserv. A total of 35 clinicians inquired about the study and were offered free participation. Of these, 26 (74%, 26/35) enrolled in the study and started the tutorial, and 22 (62%, 22/35) completed it. Furthermore, 18 (51%, 18/35) trainees participated in the live applied training session after completing the tutorial. The Allendale Institutional Review Board approved the study protocol, and all participants signed informed consent statements.

Of the 26 community clinicians starting the Web-based tutorial, 23 (88%) were female, 22 (85%) white, 1 (4%) African American, 4 (15%) Hispanic, and 3 (11%) other or mixed racial categories. Participants came from 15 US states and one each from Mexico, Brazil, Canada, and the United Kingdom. Mean age was 41.6 years (standard deviation [SD]=11.5, range: 26-63 years), and mean years of clinical experience was 10.5 (SD=7.6, range: 1-26 years). Eleven (42%, 11/26) were social workers, 11 (42%, 11/26) psychologists, 2 (8%, 2/26) marriage and family therapists, 1 (4%, 1/26) a psychiatrist, and 1 (4%, 1/26) a psychiatric nurse. Additionally, 24 (92%, 24/26) were actively conducting psychotherapy with clients. Only 3 (11%) reported having received any prior formal training in IPT: one through a continuing education workshop, one as part of undergraduate coursework, and one in graduate coursework. Three participants (14%, 3/26) reported having used some IPT techniques in their practice before participating in the study.

### Web-Based Tutorial

#### Efficacy: Improvement in Knowledge of Interpersonal Psychotherapy (IPT) Concepts

The mean number of correct answers on the 38-item IPT concepts and skills quiz improved significantly from 16.5 (SD 4.6) on pretest to 27.5 (SD 4.0) on posttest, *t*_21_=15.7, *P*<.001. The standardized effect size for the change was large: *d*=2.53, 95% CI 2.23-2.92.

#### User Satisfaction: Technical Features

**Figure 1:**
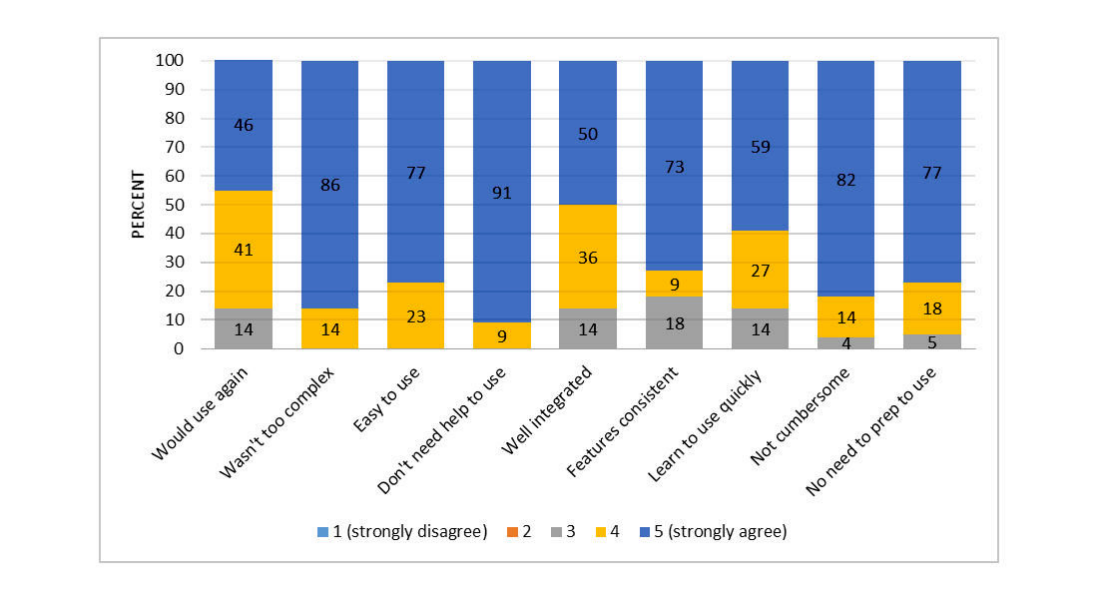
System Usability Scale ratings of user satisfaction with technical features of Web-based tutorial.

The SUS evaluated user satisfaction with the technical features of the Web-based tutorial. Mean SUS score was 90.6 (SD 11.4). This corresponds to a mean rating between “excellent” and “best imaginable.” Figure 1 shows ratings on individual SUS items. Users found the technical features of the tutorial easy to use and understand. The mean global rating on the user-friendliness item was 5.7 (between good and excellent; range: 1=worst imaginable to 7=best imaginable).

#### User Satisfaction: Clinical Content

**Figure 2:**
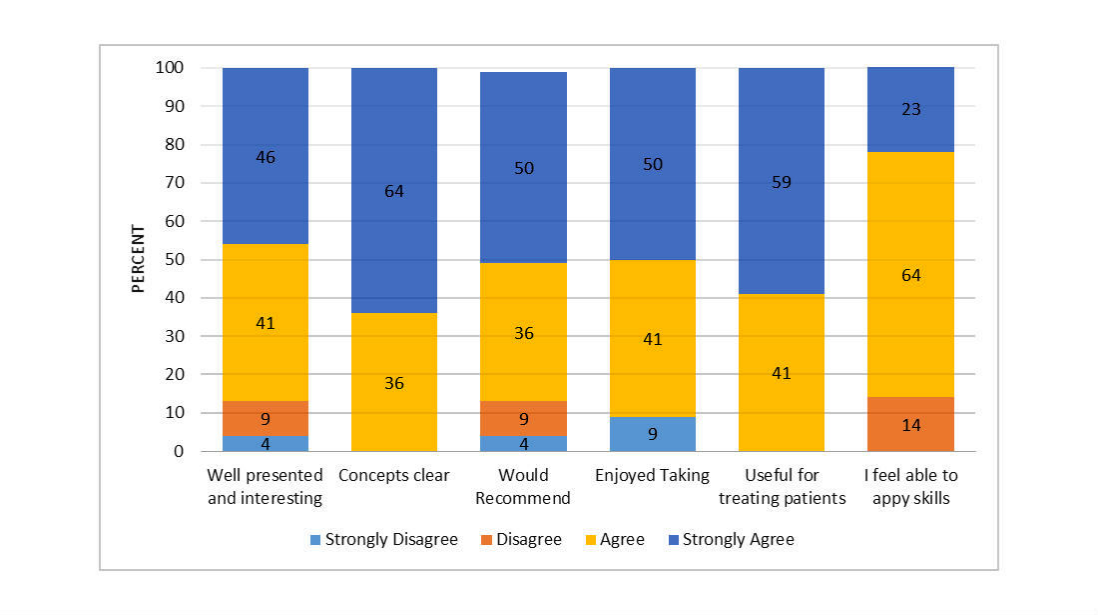
Ratings of user satisfaction: clinical content of Web-based tutorial.

Figure 2 illustrates user satisfaction with the clinical content of the tutorial. Clinicians found the concepts clearly presented in an interesting fashion and found the content useful for helping them treat depressed clients. The mean global rating of user satisfaction with the clinical content was 3.2 (range: 1=very dissatisfied to 4=very satisfied).

User satisfaction with the tutorial was also evaluated by whether clinicians felt the learning objectives of each module were met. Nineteen learning objectives were identified a priori for the seven modules (Table 1). Trainees rated whether learning objectives were met after completing each module. They rated the learning objectives as having being met 95% (18/19) of the time, on average.

Global ratings of how much the trainee learned (scale range from 1=very little to 5=a great deal) was also obtained, as required by continuing education accreditation agencies. The mean rating of trainees learning from the Web-based tutorial was 3.91.

**Figure 3:**
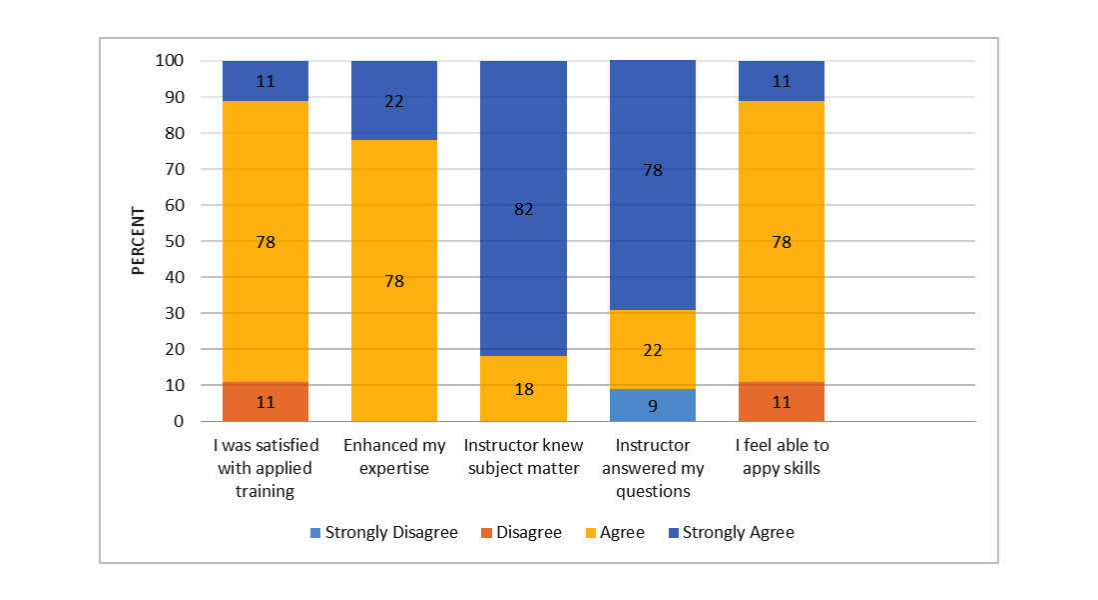
Ratings of user satisfaction: applied training via videoconference.

### Applied Training

User satisfaction with the applied training session via videoconference appears in Figure 3. Overall, trainees felt the program enhanced their professional expertise and felt able to apply the skills learned to actual treatment. Mean global rating of user satisfaction with the applied training was 3.5 (range: 1=very dissatisfied to 4=very satisfied). Mean rating of learning from the applied training was 3.94 (scale range from 1=very little to 5=a great deal).

### Web Portal

**Figure 4:**
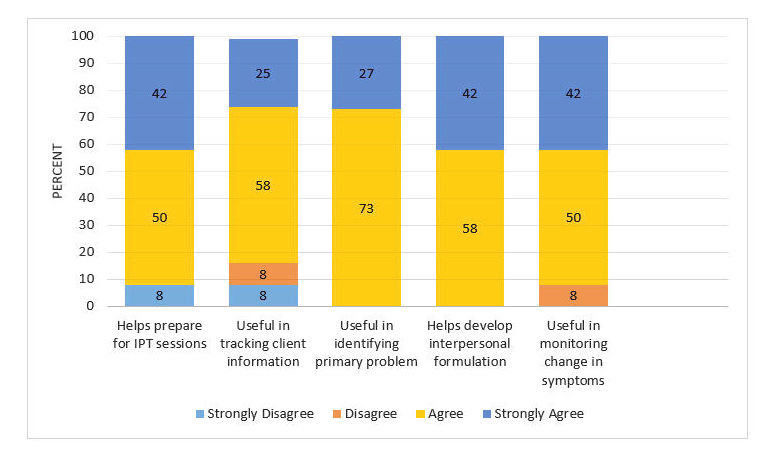
Ratings of user satisfaction: posttraining Web portal.

Figure 4 shows user satisfaction with the Web portal. Trainees thought the website helpful in preparing for IPT sessions, identifying the client’s IPT focus area, developing an interpersonal formulation, and monitoring changes in the client symptoms. Overall, satisfaction rating was 3.3 (range: 1=very dissatisfied to 4=very satisfied).

### Duration of Training

The mean time it took trainees to complete the Web-based tutorial was 3.3 hours (SD=0.8, range 2.3-5.0 hours). The average module was 29.1 min (SD 18.3). The tutorial was completed over a mean of 27.4 days (SD=22.9, range 1-66 days). The mean duration for participation in the entire training protocol (start of Web-based tutorial to evaluation of Web portal) was 91 days (SD 18.5).

## Discussion

### Principal Findings

This pilot study provides evidence to support the efficacy and feasibility of this technologically advanced, three-part therapist training intervention on IPT for major depression. Results supported both our hypotheses: the tutorial increased trainee knowledge of IPT, and trainees reported high levels of satisfaction with the three training components. User satisfaction has critical importance: if trainees do not like a training program, find it too difficult to use, or not useful, they will not complete it. In our study, trainees described high satisfaction with both technical aspects and clinical content of the training components and had a completion rate of 85% for the Web-based tutorial and 69% for the live applied training. This ranks somewhat higher than average compared with other Web-based trainings [47]. Although we could not assess the total number of possible participants who received notification of the study training opportunity, impressions from ease of recruitment further support the potential demands and interest in this vehicle (enrollment was limited to 35 participants due to budgetary constraints).

If successfully disseminated, this intervention may assist academic training programs in solving the dual challenges they face in expanding training curricula to include EBT’s and ensuring the training is effective. IPT in particular is an EBT in which most practitioners do not receive training, despite its strong empirical standing. As no treatment is universally effective, expanding training options to include multiple EBTs helps produce more well-rounded clinicians and provides more treatment options for patients, some of whom may prefer or respond better to IPT than other EBTs. IPT has been far less disseminated than CBT (which has comparable supportive evidence) and psychodynamic therapy (which has far less empirical support). IPT uses the medical model of illness which makes it very compatible with clients treated with a combination of psychotherapy and medication.

From a practical standpoint, the model this study used augments traditional approaches to training rather than replacing them [48-50]. Web-based tutorials can lay conceptual groundwork, like a textbook, but with the added advantages the technology affords in enhancing learning. Through the use of video recordings and illustrated vignettes, trainees can observe demonstrations of skills being correctly implemented in a variety of situations. Ongoing self-tests incorporated in the tutorial helps ensure and document that participants understand concepts, which has particular importance in evidence based practice. Videoconferencing can overcome logistical barriers to access to domain experts and be used for supervision, including role playing. The use of role plays with supervisors when learning new skills has been found superior to simple discussion [51]. Evaluation of clinical skills by actually observing trainees applying skills is critical to success but underutilized. In our sample, only 1 trainee reported prior live skills observation as part of their IPT training, a number consistent with the 10% rate reported in other studies [21,29]. Live observation has been found to enhance learning compared with observing videotapes of trainees [52].

The technology may also enhance posttraining supervision in several ways. Videoconferencing facilitates access to training supervisors. Access to a training supervisor following training has been found an essential ingredient of posttraining success superior to other forms of posttraining options such as peer consultation [53]. The technology facilitates ongoing case discussion following training, which is critical to ensure therapist efficacy in patients seen posttraining. The Web portal helps transfer learning from classroom to practice by providing a tool to help structure sessions, monitor application of and adherence to techniques, and monitor treatment outcome. It may also help prevent “therapist drift,” as trainees could return to the website to refresh their knowledge and for ongoing retesting and monitoring [54]. While not included as part of this study, evaluation of ongoing posttraining therapist supervision using videoconference and Web portals would be informative.

Results from this study are consistent with other studies on the use of these technologies for training clinicians on other EBTs such as CBT [29,55]. This is the first application of this technology to IPT training to be empirically evaluated that we are aware of (there has been one Web-based training pilot for Interpersonal Social Rhythms Therapy [IPSRT], an adaptation of IPT for patients with bipolar disorder [56]). Although IPT is less structured than CBT, there was no reason to think the methodology would have less success. In this study, we did not formally assess clinical competence, as the study goal was to assess user satisfaction with the methodology and improvement in conceptual knowledge. Thus, the degree of clinical competence the trainees attained is unknown. The number of live applied sessions necessary to achieve varying levels of competence in IPT using this methodology needs exploration, as does the differential impact of didactic and applied training on clinical skills. In a prior CBT study, the Web-based tutorial sufficed to improve clinical skills from poor to minimally acceptable, but the addition of four 1-hour videoconference role play sessions further improved rated clinical skills to between adequate and good [29]. Competence was assessed using role plays conducted via videoconference, which were recorded and evaluated for clinical skills by a blind rater. Such a methodology may be one way to assess competence via Web-based training. Further studies are needed to explore the relationship between the number of applied sessions and levels of competence and the relative impact of didactic and applied on-line training with IPT.

### Limitations

Limitations of this pilot study include lack of randomization and a control group, such as comparison to current standard methods of clinical instruction. The small sample size limits generalizability of results. In addition, since we did not collect patient data, it is impossible to know how the training program affected clinical practice. Although trainees received no feedback following pretesting on conceptual knowledge, there is the potential for practice effects. In the absence of a control group, it is difficult to determine the extent to which improvement in scores on the tutorial was due to training as opposed to induction bias. However, some studies have found pretests increase learning by orienting learners to subsequent information [35].

### Conclusions

Future studies could include a larger sample size, as well a cohort of recent graduates from academic internships who are interested in additional psychotherapy training following graduation. Such training could count toward continuing education credits [48]. In addition, further study of the stability of training is warranted. In summary, results support the use of these technologies in training clinicians in EBTs and warrant further study to empirically evaluate these training methods compared to and in conjunction with current methodologies.

## Appendix

Multimedia Appendix 1 Example of Web-based tutorial multimedia content.

Multimedia Appendix 2 Example of clinical vignettes and challenge questions.

Multimedia Appendix 3 Example of pre-session checklist from IPT Web portal.

Multimedia Appendix 4 Example of post-session checklist from IPT Web portal.

## Abbreviations

CBT: cognitive behavioral therapy
EBT: evidence-based therapy
IPT: interpersonal psychotherapy
SUS: System Usability Scale
