# Supplementary figures and images for: Web-Based Therapist Training in Interpersonal Psychotherapy for Depression: Pilot Study
Source: J Med Internet Res. 2017 Jul 17;19(7):e257. doi: 10.2196/jmir.7966 (PMC5537562; doi:10.2196/jmir.7966)

## Slide 1
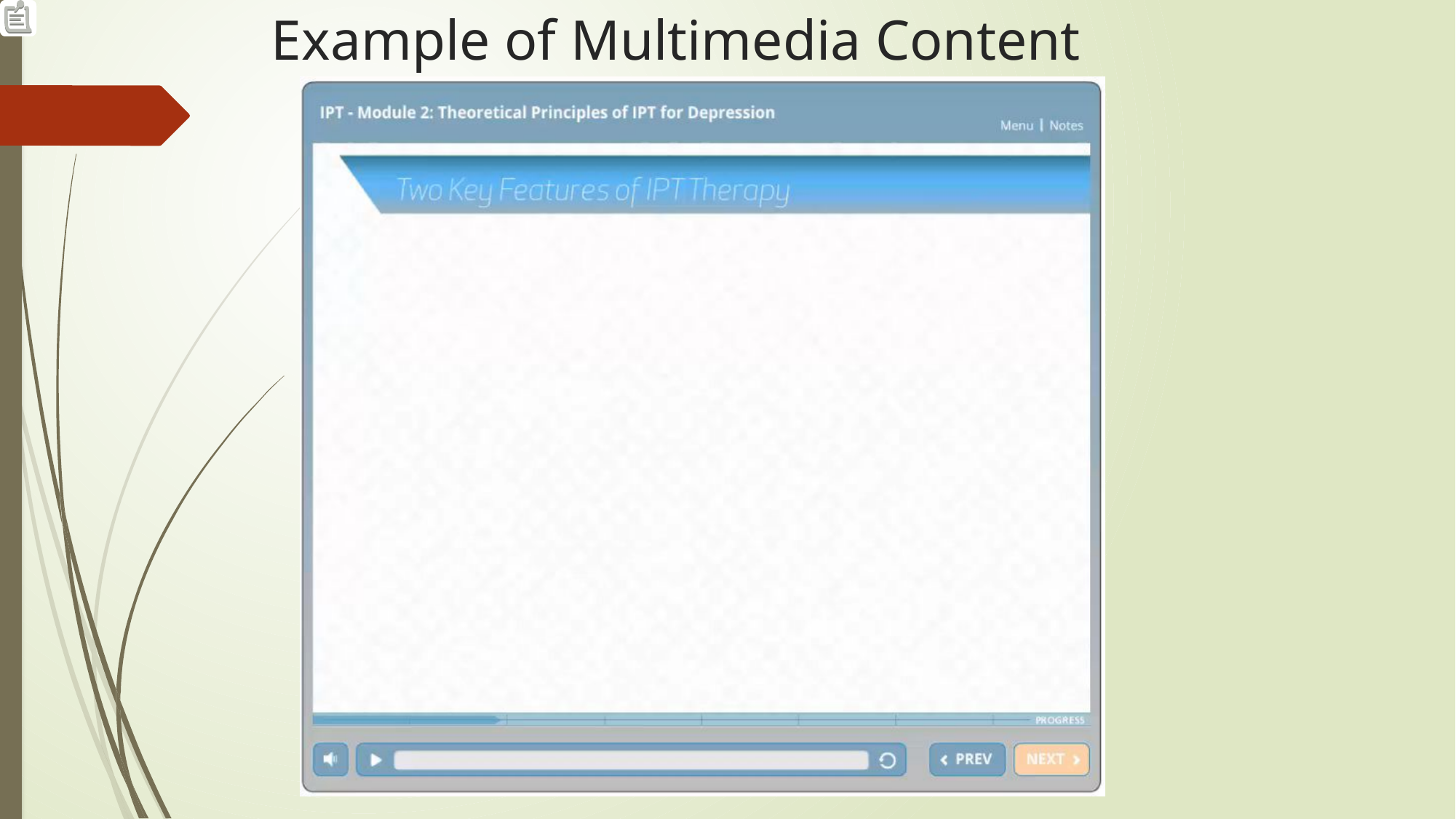

# Example of Multimedia Content

## Slide 2
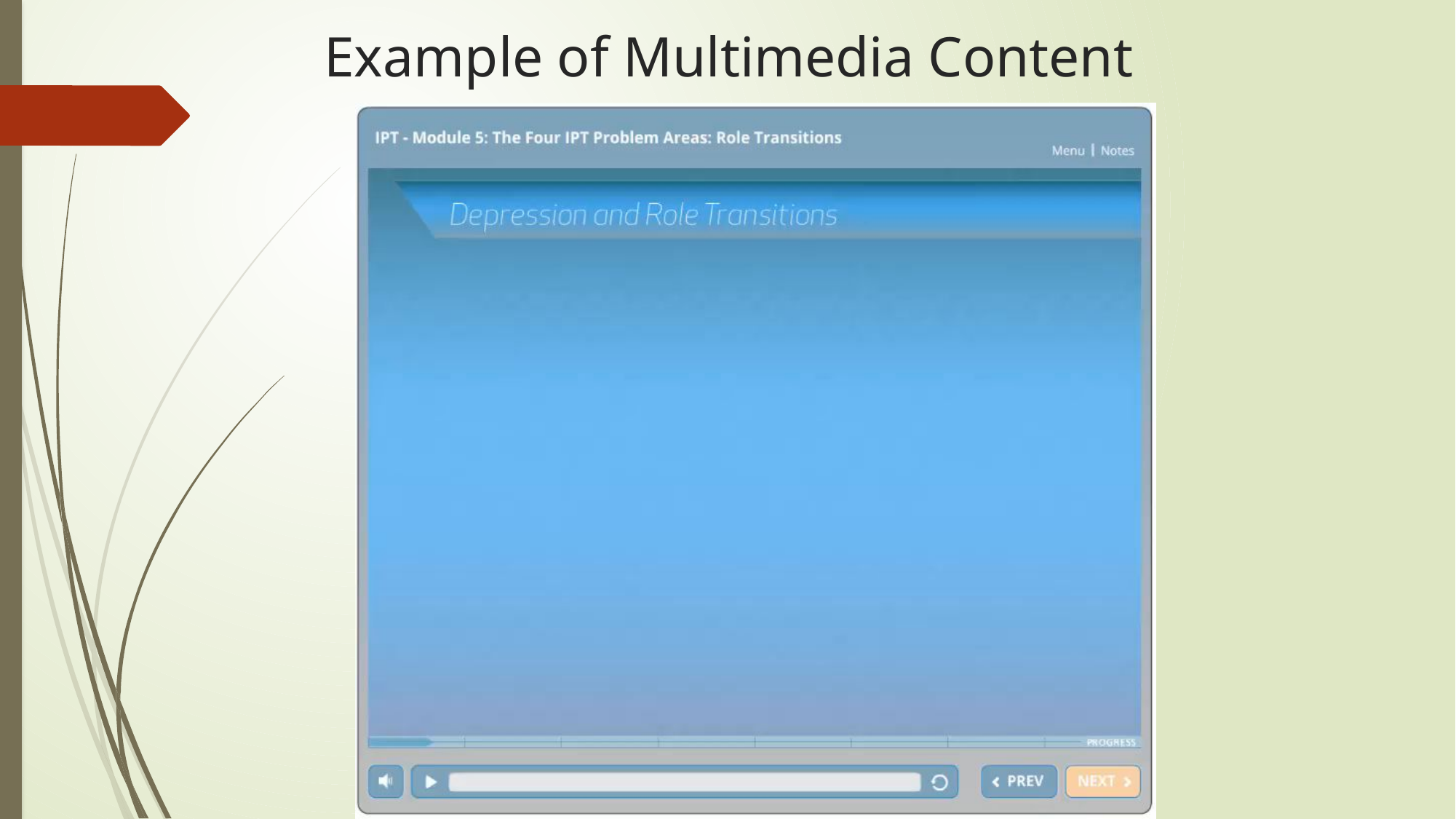

# Example of Multimedia Content

Supplement: Multimedia Appendix 1 [file jmir_v19i7e257_app1.pptx]

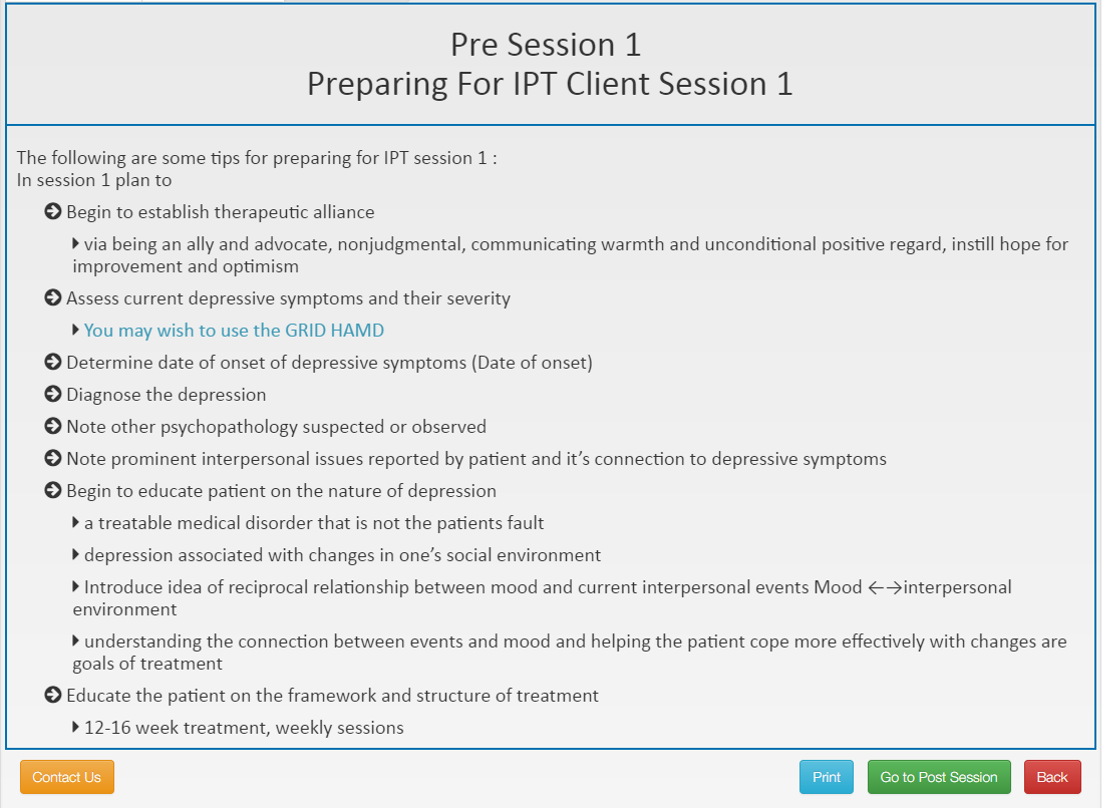

Supplement: Multimedia Appendix 3 [file jmir_v19i7e257_app3.png]

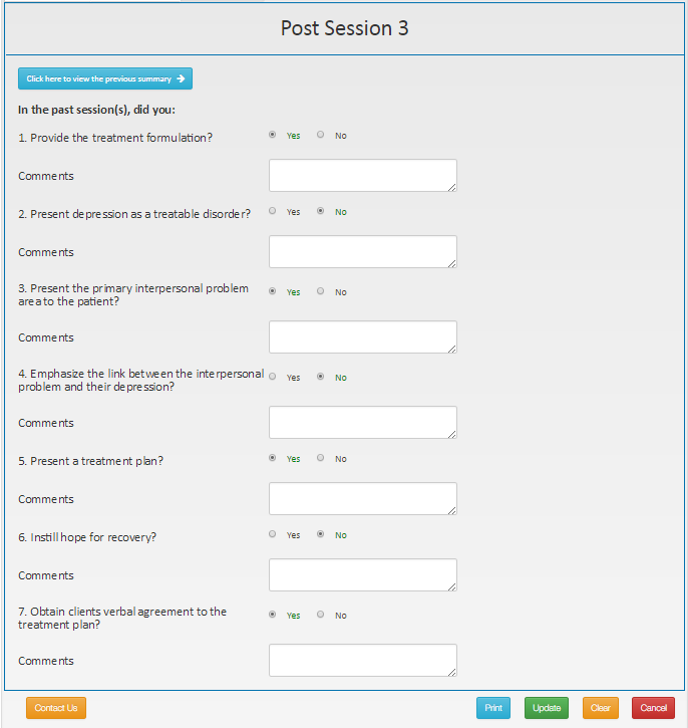

Supplement: Multimedia Appendix 4 [file jmir_v19i7e257_app4.png]
