# Supplementary material for: Web-Based Therapist Training in Interpersonal Psychotherapy for Depression: Pilot Study
Source: J Med Internet Res. 2017 Jul 17;19(7):e257. doi: 10.2196/jmir.7966 (PMC5537562; doi:10.2196/jmir.7966)

## Slide 1
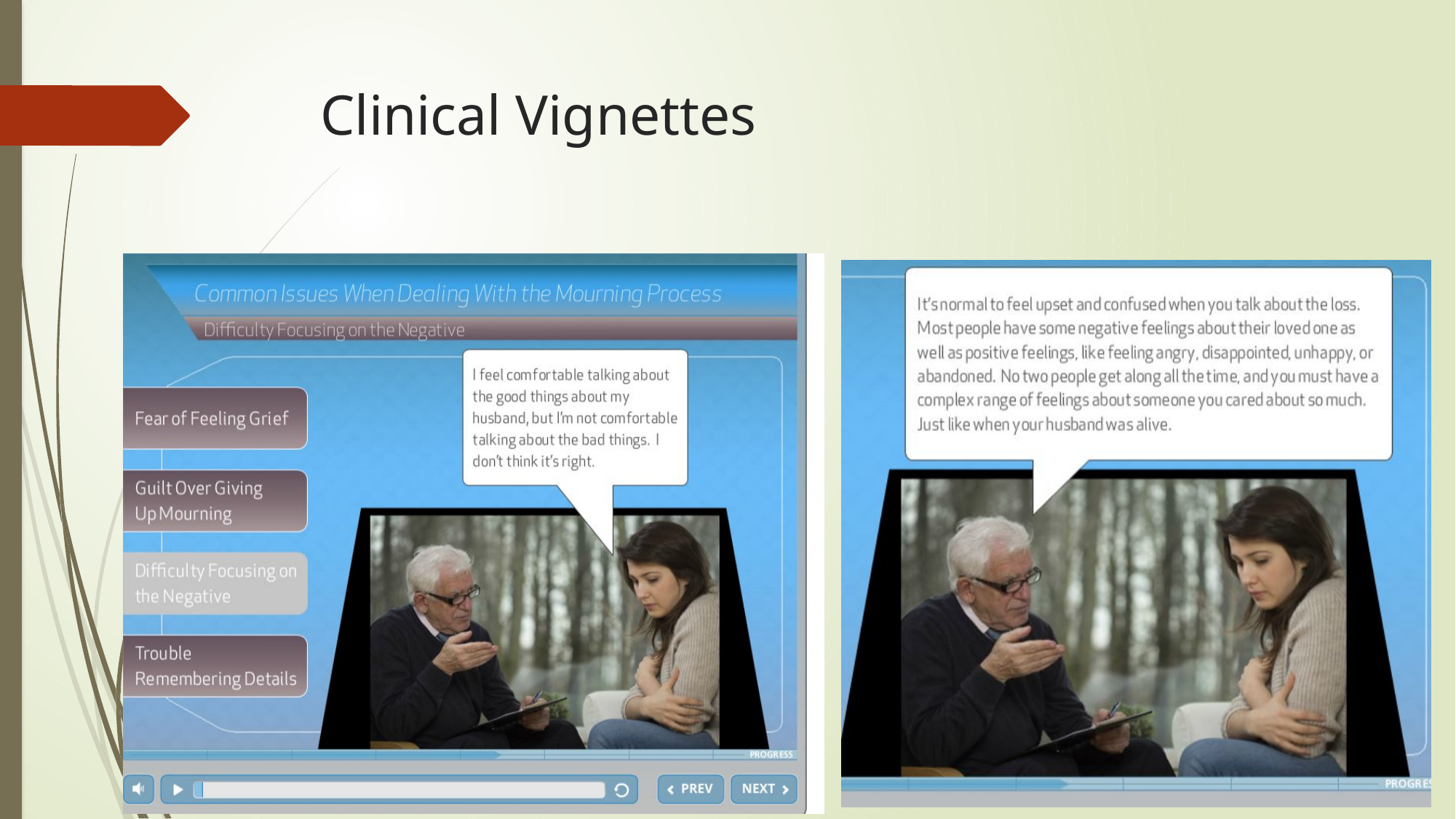

# Clinical Vignettes

## Slide 2
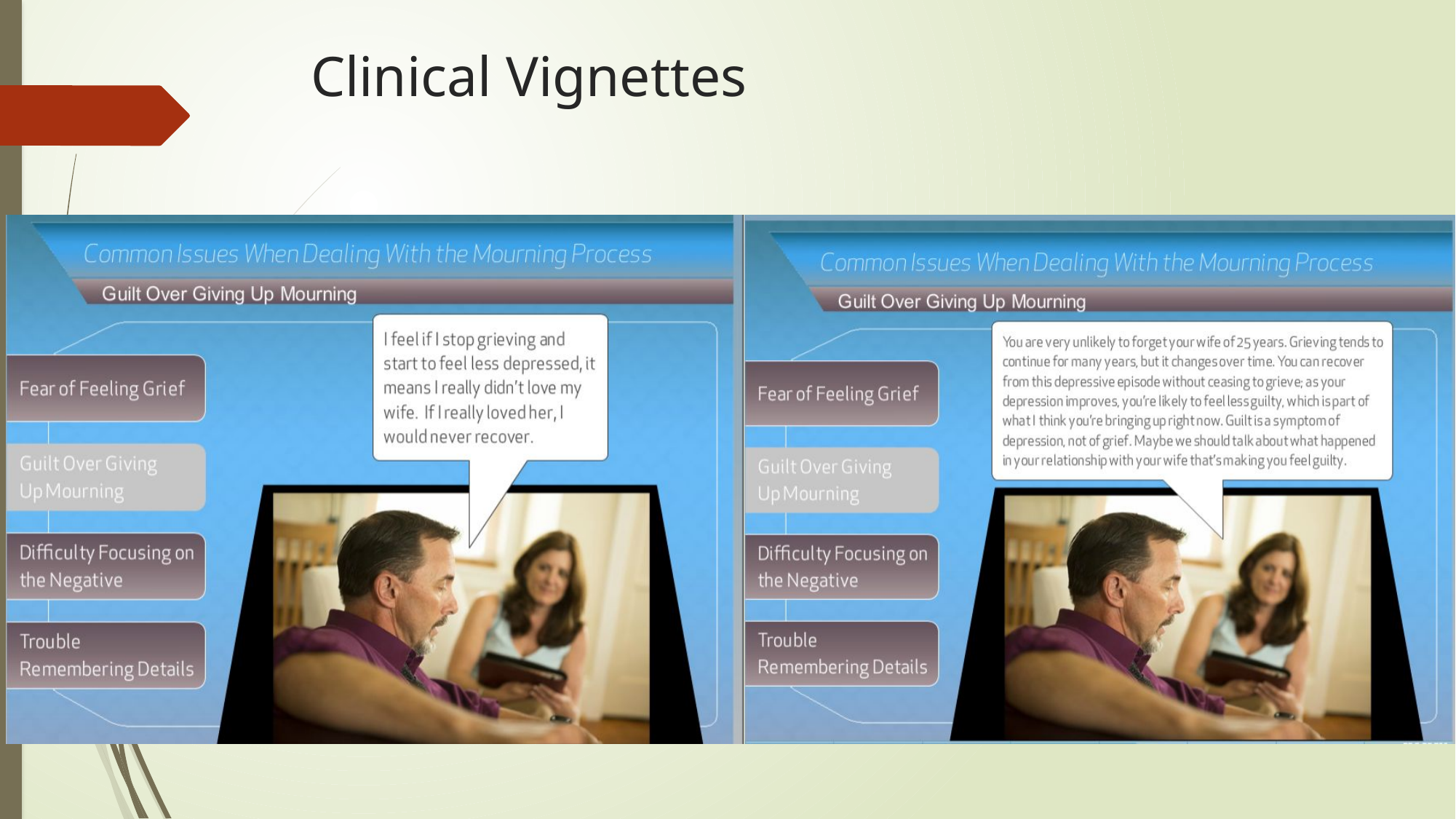

# Clinical Vignettes

## Slide 3
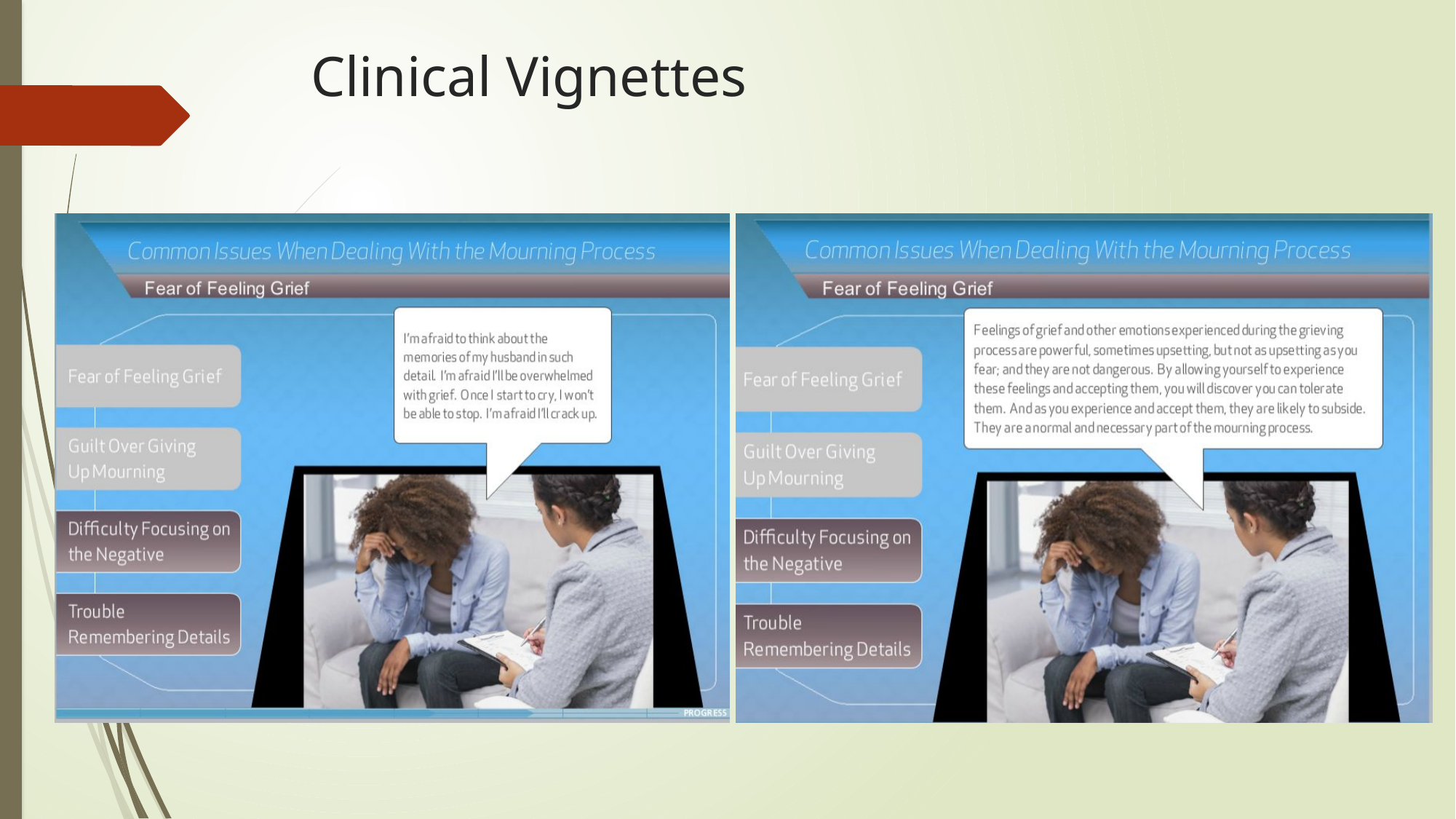

# Clinical Vignettes

## Slide 4
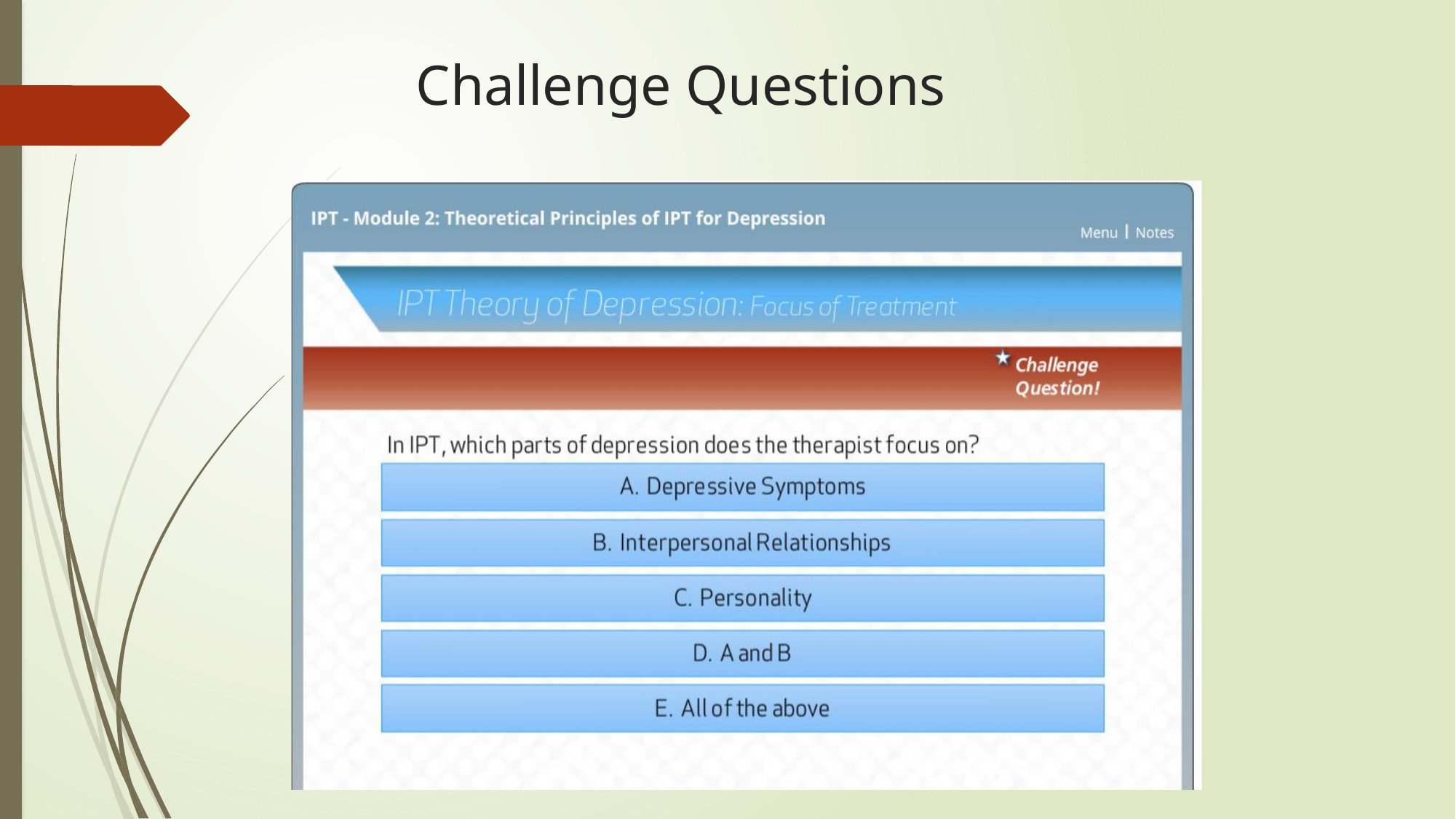

Challenge Questions

## Slide 5
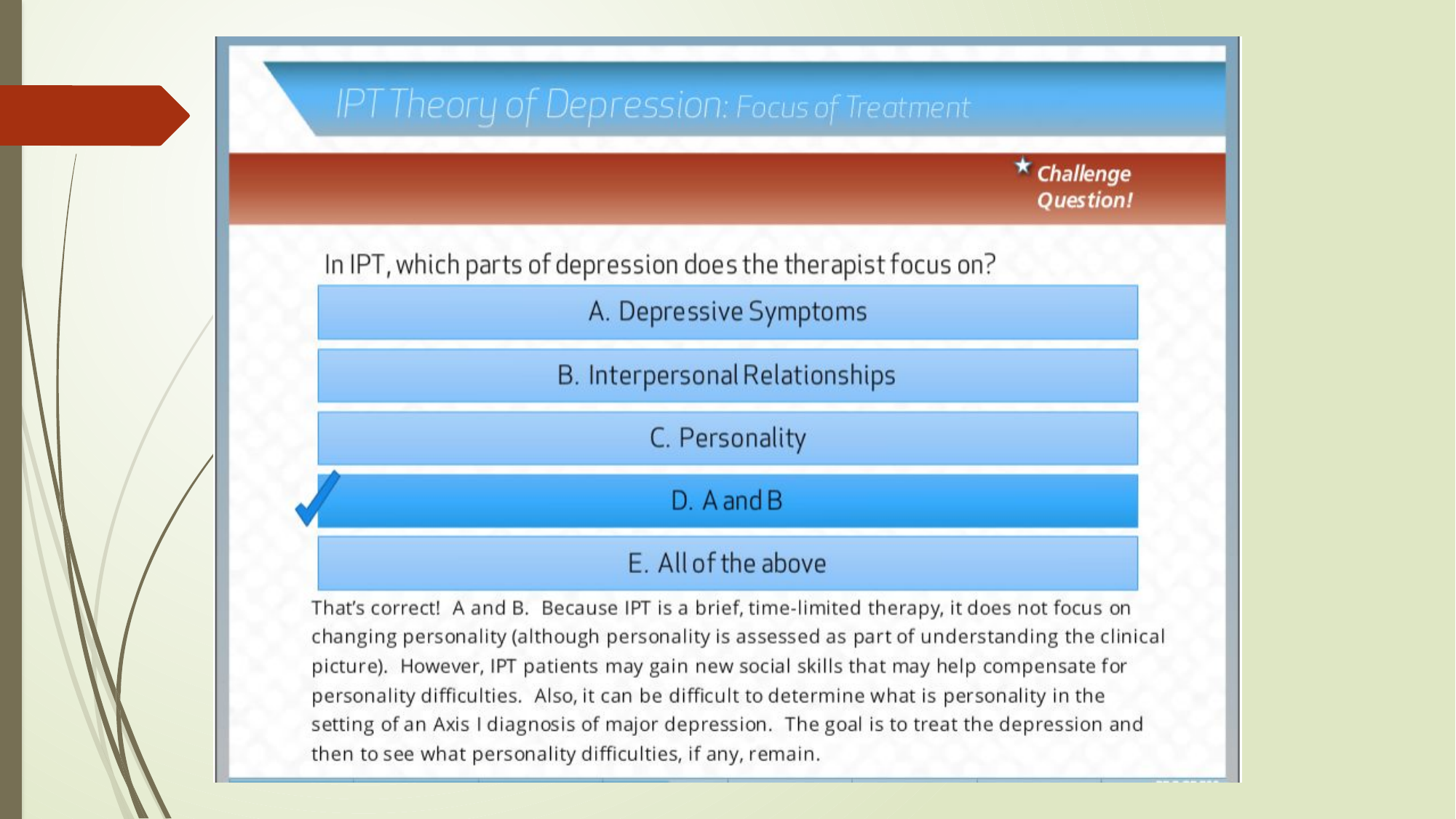

Supplement: Multimedia Appendix 2 [file jmir_v19i7e257_app2.pptx]
